# Profiles of Volatile Flavor Compounds in Milk Fermented with Different Proportional Combinations of *Lactobacillus delbrueckii* subsp. *bulgaricus* and *Streptococcus thermophilus*

**DOI:** 10.3390/molecules22101633

**Published:** 2017-09-29

**Authors:** Tong Dan, Dan Wang, Shimei Wu, Rulin Jin, Weiyi Ren, Tiansong Sun

**Affiliations:** Key Laboratory of Dairy Biotechnology and Engineering, Ministry of Education, Inner Mongolia Agricultural University, Hohhot 010018, China; dantong813218@aliyun.com (T.D.); magic136@126.com (D.W.); 15335563897@163.com (S.W.); jrl_happy@163.com (R.J.); renweiyi153@163.com (W.R.);

**Keywords:** fermented milk, volatile compounds (VOCs), solid phase microextraction (SPME), gas chromatography-mass spectrometry (GC-MS)

## Abstract

*Lactobacillus delbrueckii* subsp. *bulgaricus* and *Streptococcus thermophilus* are key factors in the fermentation process and the final quality of dairy products worldwide. This study was performed to investigate the effects of the proportions of *Lactobacillus delbrueckii* subsp. *bulgaricus* and *Streptococcus thermophilus* isolated from traditionally fermented dairy products in China and Mongolia on the profile of volatile compounds produced in samples. Six proportional combinations (1:1, 1:10, 1:50, 1:100, 1:1000, and 1:10,000) of *L. delbrueckii* subsp. *bulgaricus* IMAU20401 to *S. thermophilus* ND03 were considered, and the volatiles were identified and quantified by solid-phase microextraction and gas chromatography–mass spectrometry (SPME-GC-MS) against an internal standard. In total, 89 volatile flavor compounds, consisting of aldehydes, ketones, acids, alcohols, esters, and aromatic hydrocarbons, were identified. Among these, some key flavor volatile compounds were identified, including acetaldehyde, 3-methylbutanal, acetoin, 2-heptanone, acetic acid, butanoic acid, and 3-methyl-1-butanol. The of *L. delbrueckii* subsp. *bulgaricus* IMAU20401 to *S. thermophilus* ND03 influenced the type and concentration of volatiles produced. In particular, aldehydes and ketones were present at higher concentrations in the 1:1000 treatment combination than in the other combinations. Our findings emphasize the importance of selecting the appropriate proportions of *L. delbrueckii* subsp. *bulgaricus* and *S. thermophilus* for the starter culture in determining the final profile of volatiles and the overall flavor of dairy products.

## 1. Introduction

*Lactobacillus delbrueckii* subsp. *bulgaricus* and *Streptococcus thermophilus* are lactic acid bacteria (LAB) that have been isolated from a variety of habitats, particularly traditionally fermented food [1,2]. They play important roles in the production of dairy products, particularly yogurt, where they are the keys to final product quality. *Lactobacillus delbrueckii* subsp. *bulgaricus* and *S. thermophilus* used in mixed culture have a symbiotic relationship in milk due to the exchange of metabolites [3]. For example, *L. delbrueckii* subsp. *bulgaricus* can easily utilize the pyruvic acid, formic acid, folic acid, and long-chain fatty acids produced by *S. thermophilus*, whereas the peptides, free amino acids, and putrescine produced by *L. delbrueckii* subsp. *bulgaricus* stimulate the growth of *S. thermophilus* [4,5].

Yogurt production is perhaps one of the most complex milk fermentation processes; milk fermentation with *L. delbrueckii* subsp. *bulgaricus* and *S. thermophilus* produces yogurt with good flavor, acidity, and viscosity. Flavor is achieved by the integration of a variety of volatile compounds (VOCs), including acids, aldehydes, ketones, alcohols, esters, and hydrocarbons. These compounds can impart favorable flavors to yogurt [6,7]. Using solid-phase microextraction coupled with gas chromatography–mass spectrometry (SPME-GC-MS), Settachaimongkon et al. identified VOCs produced in milk by *L. delbrueckii* subsp. *bulgaricus* and *S. thermophilus*, including fatty acids, alcohols, and sulfur compounds [8]. Dan et al. evaluated the VOCs produced by *L. delbrueckii* subsp. *bulgaricus* and *S. thermophilus* from traditional fermented milk and reported similar results; these VOCs included acids, alcohols, aldehydes, ketones, and hydrocarbons [9].

The introduction of GC-MS has accelerated the field of flavor chemistry, especially when linked to SPME as a pretreatment method [10,11]. The main advantages of SPME are its simplicity, low cost, ease of automation, and in situ sampling [12]. SPME coupled with GC-MS has been used widely to evaluate the flavor chemical profiles of volatile aromas produced by a wide variety of substances, including fermented milk [13,14], the fruit and sap of mango cultivars [15], grapes and wine [16], dry fermented sausage [17], and alcoholic beverages [18].

*Lactobacillus delbrueckii* subsp. *bulgaricus* IMAU20401 and *S. thermophilus* ND03 were isolated from traditional fermented dairy products in China and Mongolia and selected based on their excellent processing properties, such as flavor, acidity, viscosity, and water-holding capacity [9,19]. This study was performed to quantify variations in the profile of volatiles produced using different proportional combinations of *L. delbrueckii* subsp. *bulgaricus* IMAU20401 and *S. thermophilus* ND03.

## 2. Results and Discussion

### 2.1. Microbiological Counts

The viable counts of the different proportional combinations of *L. delbrueckii* subsp. *bulgaricus* IMAU20401 and *S. thermophilus* ND03 (1:1, 1:10, 1:50, 1:100, 1:1000, and 1:10,000) in the samples were 5 × 10^9^, 4 × 10^9^, 2.8 × 10^9^, 3.3 × 10^9^, 1.60 × 10^9^, and 0.09 × 10^9^ CFU/mL^−1^, respectively, at pH 4.5.

### 2.2. Extraction Temperature and Time Effect

**Figure 1:**
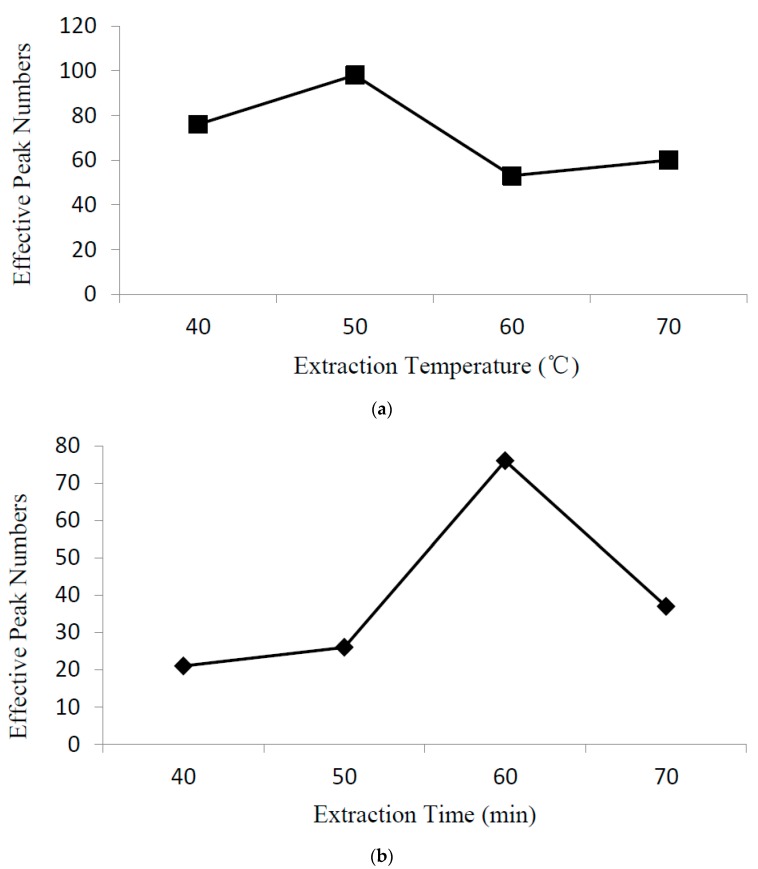
Effects of extraction temperature and time on extraction efficiency.

A study to optimize extraction conditions, including the sample temperature and extraction time of volatile compounds present in samples, was performed using SPME fiber (50/30 μm DVB/Carboxen/PDMS). The sample temperature and extraction time are important parameters in the SPME sampling process and can increase the extraction efficiency when optimized. The sample temperature and extraction time are discussed for the 1:1 proportional combination of *L. delbrueckii* subsp. *bulgaricus* IMAU20401 and *S. thermophilus* ND03. Extraction was compared at temperatures of 40 °C, 50 °C, 60 °C, and 70 °C. The effective peak numbers were enhanced at temperatures up to 50 °C and then began to decline (Figure 1a). Therefore, a temperature of 50 °C was used to study extraction time. Using extraction times ranging from 40 to 70 min, the effective peak numbers were enhanced as time increased up to 60 min and then began to decline (Figure 1b). Therefore, the optimum extraction conditions were 50 °C for 60 min.

### 2.3. Volatile Composition of Samples

**Figure 2:**
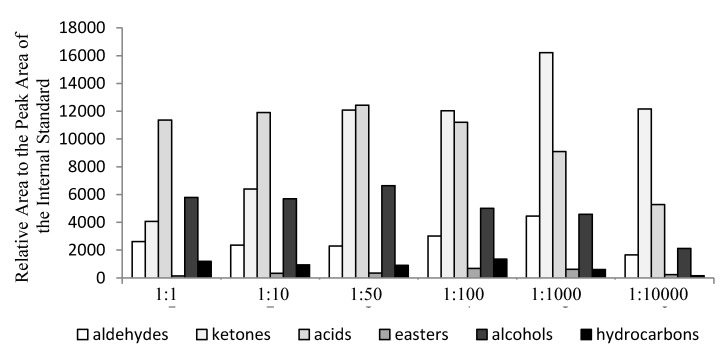
The categories and relative peak areas compared to those of the internal standards for the volatiles in milk fermented with different proportions of *L. delbrueckii* subsp. *bulgaricus* IMAU20401 and *S. thermophilus* ND03.

Figure 2 shows the categories and relative peak areas compared to those of the internal standards for the volatiles in milk fermented with different proportions of *L. delbrueckii* subsp. *bulgaricus* IMAU20401 and *S. thermophilus* ND03. The relative peak areas to those of the internal standards of aldehyde and ketone compounds were higher for 1:1000 treatment than for the other combinations, reaching 4456 and 16,219, respectively. With 1:50 treatment, the relative peak areas to those of the internal standards for acids, esters, and alcohols were higher than in the other combinations and reached 12,430, 4435, and 6633, respectively, and then began to decline. These observations indicated that smaller initial proportions of *L. delbrueckii* subsp. *bulgaricus* in samples result in a reduction of post-acidification. In contrast, the relative peak areas to those areas of internal standards for aromatic hydrocarbons were higher with 1:100 treatment than with the other combinations, reaching a value of 1353. This observation indicated that there are significant differences between the samples fermented with different proportions of *L. delbrueckii* subsp. *bulgaricus* IMAU 20,401 and *S. thermophilus* ND03 in terms of the composition of volatile compounds.

### 2.4. Profiles of Aldehyde Compounds

Volatile compounds detected using SPME pretreatment combined with GC-MS included 16 aldehyde compounds (Table 1)*.* Acetaldehyde is the key aroma compound in fermented milk products and can improve flavor [20]. *Lactobacillus delbrueckii* subsp. *bulgaricus* IMAU20401 and *S. thermophilus* ND03 are known to play important roles in producing aromatic compounds in fermented milk [9,19]. Acetaldehyde can be produced directly from ethanol by the activity of alcohol dehydrogenase as an intermediate in the metabolism of sugar [21,22]. Several metabolic pathways in *L. delbrueckii* subsp. *bulgaricus* and *S. thermophiles* have been reported to result in the production of acetaldehyde in fermented milk [21]. For example, threonine is readily converted to acetaldehyde by catalysis of threonine aldolase [23,24,25]. In this study, high levels of acetaldehyde were present in all combinations of *L. delbrueckii* subsp. *bulgaricus* IMAU20401 and *S. thermophilus* ND03, particularly 1:1000 treatment. Similar results were reported by Hamdan et al. (1973), who reported that a more abundant *S. thermophiles* population stimulated the production of aldehyde compounds in yogurt [26].

3-Methylbutanal is a branched-chain aldehyde compound derived from isoleucine and leucine by the action of enzymes [27]. As this aldehyde compound has a low taste threshold (1.2 μg /L), trace concentrations can be important characteristics in fermented milk [28,29]. In this study, a high concentration of 3-methylbutanal was present in the volatile fraction of milk fermented by all combinations of *L. delbrueckii* subsp. *bulgaricus* IMAU20401 and *S. thermophilus* ND03, particularly in the 1:100 and 1:1000 treatments, where the relative concentrations reached 8.23 and 7.8 μg/L, respectively.

Hexanal and heptanal are also important aroma compounds that contribute to good flavor in fermented milk products [23]. In this study, hexanal concentration reached 5.2 μg/L in the 1:1000 combination; the levels reached 1.43, 1.29, 2.51, and 1.24 μg/L in the 1:1, 1:10, 1:50, 1:100, and 1:10,000 combinations, respectively. As an important flavor compound, hexanal is derived from the oxidation of unsaturated fatty acids and is regularly reported in dairy products, such as fermented milk [30]. In this study, the concentration of heptanal ranged from 1.55 to 2.23 μg/L in the different combinations of *L. delbrueckii* subsp. *bulgaricus* IMAU20401 and *S. thermophilus* ND03; there was no detectable heptanal only in the 1:10,000 combination, which may have been due to the smaller initial proportions of *L. delbrueckii* subsp. *bulgaricus* in the samples. Similar results were reported by Dan et al. (2017), who reported that heptanal was produced at high levels by *L. delbrueckii* subsp. *bulgaricus* during storage [9].

The concentrations of 3-hydroxybutanal and (*E*)-2-pentenal ranged from 1.16 to 6.96 and from 1.27 to 5.44 μg/L, respectively, reaching maximum values in the 1:1000 combination. Although 3-hydroxybutanal and (*E*)-2-pentenal had lower threshold values of 27 and 1.2 μg/L, respectively, only the level of (*E*)-2-pentenal in samples was above the limit of detection. In addition to the above-mentioned aldehyde compounds, the concentrations of (*E*)-2-heptenal, (*E*,*E*)-2,4-heptadienal, (*E*)-2-nonenal, and (*E*)-2-hexenal reached maximum values in the 1:1 and 1:50 treatment combinations, respectively. These observations were similar to the results of Ning et al., (2011), who observed changes in (*E*)-2-heptenal, (*E*,*E*)-2,4-heptadienal, and (*E*)-2-nonenal in fermented camels’ milk, and of Fortini et al. who reported changes in levels of (*E*)-2-hexenal in olive oil [14,31].

### 2.5. Profiles of Ketone Compounds

In total, 13 ketone compounds were identified in the samples fermented with different proportions of *L. delbrueckii* subsp. *bulgaricus* IMAU20401 and *S. thermophilus* ND03 (Table 1). Among these, some are important flavor VOCs and were present at relatively high levels: 2,3-butanedione, 2-pentanone, 3-methyl-2-butanone, acetoin, 2-heptanone, and 2-nonanone. 2,3-Butanedione and acetoin are common metabolic products of citrate metabolism and contribute positively to the perception of buttery and creamy flavors in dairy products [32,33]. The concentration of acetoin was generally higher than those of the other ketone compounds, particularly in the 1:1000 combination. This was similar to the results reported by Rincondelgadillo et al. [34]. 2-Pentanone and 3-methyl-2-butanone were detected in all samples with concentrations of 1.67–5.27 and 1.65–5.28 μg/L, respectively, peaking in the 1:1000 combination. Similarly, the values for 2-heptanone and 2-nonanone reached 25.56 and 10.67 μg/L, respectively; in particular, the 2-nonanone concentration was above the detection limit (5 μg/L) in the 1:1000 combination. These compounds have frequently been reported in dairy products, including milk, fermented milk, and cheese [11,35,36].

### 2.6. Profiles of Acid Compounds

Sixteen acid compounds were identified in the different combinations of *L. delbrueckii* subsp. *bulgaricus* IMAU20401 and *S. thermophilus* ND03 (Table 1)*.* Hexanoic acid is a saturated fatty acid with six carbons and one carboxylic group [37]. This acid compound is an important aroma compound that has been detected frequently in a number of different dairy products [14,38]. In this study, hexanoic acid was the most abundant acid compound and was found in all treatment combinations at concentrations ranging from 22.88 to 40.57 μg/L. The next most abundant was acetic acid, followed by butanoic acid and octanoic acid. Acetic acid is the main subproduct of LAB fermentation [39,40]. In this study, acetic acid concentrations ranged from 7.89 to 24.65 μg/L, with the highest value of 24.65 μg/L seen in the 1:1 treatment combination. Rincon-Delgadillo et al. reported similar results [34]. In the present study, high levels of butanoic acid and octanoic acid were detected in all treatment combinations and ranged from 0.62 to 16.83 and from 6.08 to 13.43 μg/L, respectively. These acid compounds are frequently found in dairy products, including fermented milk [33].

3-Methylbutanoic acid and its corresponding alcohols (3-methylbutanol) and aldehydes (3-methylbutanal) are amino acid degradation products. 3-Methylbutanal can be converted to 3-methylbutanoic acid via oxidation [40]. In this study, 3-methylbutanoic acid concentrations ranged from 3.35 to 8.33 μg/L, peaking in the 1:50 treatment combination. The concentrations of 2-methylhexanoic acid, 2-methylbutanoic acid, and pentanoic acid were 2.74–8.15, 7.16–12.42, and 8.38–11.86 μg/L, respectively. These values also peaked in the 1:50 treatment combination.

Low levels of heptanoic acid, nonanoic acid, 2-undecenoic acid, and *n*-decanoic acid were also detected at concentrations of 1.04–2.02, 1.69–2.99, 0.35–1.11, and 1.07–3.79 μg/L, respectively. Although these concentrations were relatively low compared with those of the other acid compounds, they have also been reported frequently in dairy products by other groups [14,16,23].

### 2.7. Profiles of Ester Compounds

Esterification reactions occur during lactose fermentation or amino acid catabolism [41]. These ester compounds have low taste thresholds, and therefore low levels can still contribute to good flavor in fermented dairy products. The formation of ester compounds has been studied in dairy products, and some ester compounds were detected in milk fermented by lactococci [32]. Six ester compounds were identified in the present study (Table 1). Among these, traces of formic acid hexyl ester were detected in all treatment combinations, particularly in the 1:100 treatment, where the concentration reached 2.65 μg/L, which was higher than those in the other treatment combinations.

### 2.8. Profiles of Alcohol Compounds

Similar to ketone compounds, alcohol compounds are important for flavor in dairy products [8]. In general, alcohol compounds are generated by reduction from the corresponding aldehydes [27]. In total, 25 alcohol compounds were detected in the present study (Table 1). High levels of 3-methylbutanol, 1-hexanol, and 1-heptanol were detected in all samples, with concentrations of 14.89–23.12, 3.05–16.29, and 1.86–12.49 μg/L, respectively. The concentrations of these alcohol compounds were higher in the 1:1, 1:10, and 1:50 treatment combinations compared with in the other treatments. 3-Methylbutanol is present in relatively large quantities in dairy products and imparts an “alcoholic and floral” flavor to fermented milk [41]. In the present study, 3-methylbutanol concentrations were calculated in the 1:10 treatment combination and showed a maximum value of 23.12 μg/L. 1-Hexanol and 1-heptanol have also been reported in dairy products [9,14,42]. In the present study, their concentrations reached 16.29 and 12.49 μg/L, respectively, in the 1:1 treatment combination.

Methylalcohols can be generated from corresponding methylketones by reductase activity. Six methylalcohols were detected in the different treatment combinations: 3-methyl-2-butanol, 3-methyl-3-buten-1-ol, 2-methyl-3-pentanol, 3-methyl-2-heptanol, 3-methyl-2-hexanol, and 5-methyl-2-heptanol. Among these, high levels of 3-methyl-2-heptanol were detected, reaching 12.14 μg/L in the 1:50 treatment combination.

2-Heptanol and 2-nonanol have been reported previously at relatively high concentrations in dairy products [43]. However, only low levels of 2-heptanol and 2-nonanol were detected in the present study: the 2-heptanol concentration varied between 0.42 and 0.93 μg/L in the 1:10 and 1:10,000 treatment combinations, respectively, and the 2-nonanol concentration ranged from 1.14 to 3.98 μg/L in the 1:1, 1:10, 1:50, and 1:100 treatment combinations.

1-Octen-3-ol has a low threshold value (1.5 μg/L) and contributes to good flavor in dairy products, even at low concentrations [41]. In the present study, 1-octen-3-ol was present at levels above the detection limit, although it was present only in the 1:1 treatment combination. Friedrich and Acree reported this alcohol compound previously [44].

### 2.9. Profiles of Aromatic Hydrocarbons

Aromatic hydrocarbons consist of a wide variety of natural and synthetic low molecular mass compounds [45]. Thirteen aromatic hydrocarbons were detected in the present study (Table 1). Among these, a number of important aromatic hydrocarbons were found in the 1:10, 1:50, and 1:1000 treatment combinations, including toluene, 2,4-dimethylhexane, and (*Z*)-2-heptene, at concentrations of 0.23–5.13, 1.13–7.39, 0–4.84 μg/L, respectively. Toluene has been detected previously in fermented milk using the SPME technique [9,36]. Although high levels of 2,4-dimethylhexane and (*Z*)-2-heptene were detected in the present study, the influence of these compounds on the flavor of samples is not clear. Future work will target these aromatic hydrocarbons by examining samples to obtain a better understanding of their roles in fermented milk.

### 2.10. Principal Component Analysis of Volatile Compounds

**Figure 3:**
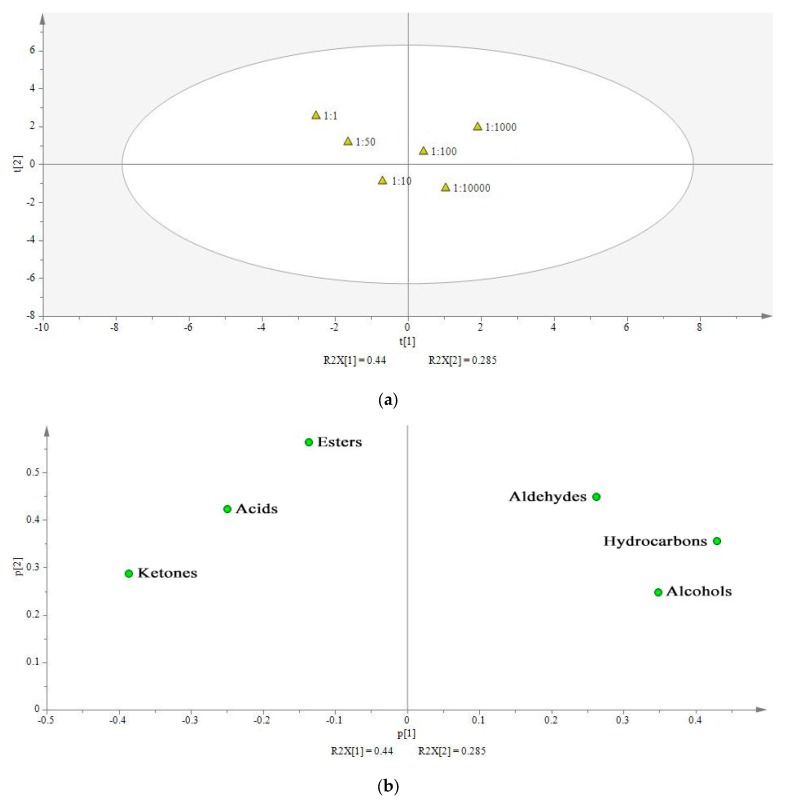
Principal component analysis (PCA) analysis: (**a**) Score scatter plot of six groups of samples with different strain ratios; (**b**) Loading scatter plot of six classes of volatile components.

Principal component analysis (PCA) was performed to examine the differences in volatile compounds from milk fermented with different proportions of *L. delbrueckii* subsp. *bulgaricus* IMAU20401 and *S. thermophilus* ND03. Figure 3a indicates the score scatter plot for six treatments (1:1, 1:10, 1:50, 1:100, 1:1000, and 1:10,000). The samples could be divided into two distinctive groups. The first group included treatments with a lower proportion of *L. delbrueckii* subsp. *bulgaricus* IMAU20401 in the samples (1:100, 1:1000, and 1:1000), whereas the second group included treatments with a higher proportion of *L. delbrueckii* subsp. *bulgaricus* IMAU20401 in the samples (1:1, 1:10, 1:50). The results revealed differences in flavor compounds between the two groups. Figure 3b shows a loading scatter plot of six classes of volatile components. There was a positive correlation between flavor and aldehyde compounds in the 1:1000 treatment combination. Nevertheless, a negative correlation was observed between flavor and acid compounds in the 1:1 treatment combination. These results also indicated that the proportions of *L. delbrueckii* subsp. *bulgaricus* and *S. thermophilus* influence the flavor of samples.

## 3. Experimental

### 3.1. Bacterial Isolates and Reagents

*Lactobacillus delbrueckii* subsp. *bulgaricus* IMAU20401 was originally isolated from yogurt collected in Huvsgel province, Mongolia; *S. thermophilus* ND03 was isolated from *kurut* collected in Qinghai province, China. C3–C25 *n*-alkanes were purchased from Accustandard (New Haven, CT, USA). 1,2-Dichlorobenzene (internal standard, ISTD) was obtained from Sigma-Aldrich (Steinheim, Germany). de Man Rogosa Sharpe (MRS) and M17 broths were acquired from OXOID (Hampshire, UK). Whole milk powder was purchased from NZMP Ltd. (Wellington, New Zealand). Furthermore, acetaldehyde (from Dr. Ehrenstorfer), acetic acid (from Dr. Ehrenstorfer), lactic acid (from Sigma-Aldrich), 2,3-butanedione (from Sigma-Aldrich), and acetoin (from Sigma-Aldrich) were also used as standards to confirm identifications.

### 3.2. Sample Production

Whole milk powder was mixed with water at 50 °C to a total solids content of 11.5 g/100 g and supplemented with 6.5 g/100 g of sucrose. The prepared medium was stored at 4 °C prior to use.

Frozen cells of *L. delbrueckii* subsp. *bulgaricus* IMAU20401 and *S. thermophilus* ND03 were activated by three subcultures in MRS broth and then inoculated into the milk/sucrose medium. Six different inoculation ratios were used: 1:1, 1:10, 1:50, 1:100, 1:1000, and 1:10,000 of *L. delbrueckii* subsp. *bulgaricus* IMAU20401 to *S. thermophilus* ND03. In all combinations, *S. thermophilus* ND03 was inoculated at a concentration of 5 × 10^7^ colony-forming units (CFU)/mL. After inoculation, fermentation was allowed to proceed at 42 °C until the pH fell to 4.5. Samples were taken and stored at −20 °C prior to analysis of the volatile compounds.

### 3.3. Microbiological Counts

Viable bacterial counts in the different proportional combinations of *L. delbrueckii* subsp. *bulgaricus* IMAU20401 and *S. thermophilus* ND03 (1:1, 1:10, 1:50, 1:100, 1:1000, and 1:10,000) in samples were recorded at pH 4.5 by plating on MRS agar and incubating at 37 °C for 48 h.

### 3.4. HS-SPME-GC-MS Analysis

The headspace solid-phase microextraction–gas chromatography-mass spectrometry (HS-SPME-GC-MS) technique was used to analyze the volatile compounds produced in each of the combinations of *L. delbrueckii* subsp. *bulgaricus* IMAU20401 to *S. thermophilus* ND03 according to the methods of Aunsbjerg et al. [46]. Briefly, as ISTD, 1,2-dichlorobenzene and 5 mL of sample were mixed in 20-mL glass vials (CNW Technologies, Germany) fitted with a PTFE/silicone septum. The final concentration of ISTD in each sample was 10 μg/L. The samples were stirred for 5 min at 50 °C using microstirring bars to allow the samples to reach equilibrium. Subsequently, a SPME fiber (50/30 um DVB/Carboxen/PDMS; Supelco, Inc. Bellefonte, PA, USA) was exposed in the headspace for 60 min under the same conditions; the fiber was then immediately inserted into the injection port of a 7890 B GC (Agilent Technologies, Inc., Palo Alto, CA, USA) for 5 min at 270 °C to desorb volatile compounds into the GC. The optimum extraction conditions were selected based on preliminary experiments on SPME extraction of samples (1:1 proportional combinations of *L. delbrueckii* subsp. *bulgaricus* IMAU20401 and *S. thermophilus* ND03) at different extraction temperatures (40 °C, 50 °C, 60 °C, and 70 °C) and for different times (40, 50, 60, and 70 min).

### 3.5. Identification of Volatile Compounds

The volatile compounds from each combination were identified using a 7890 B GC equipped with a 5977 A mass-selective detector (MSD; both Agilent Technologies, Inc.) equipped with an HP-5MS column (length, 30 m; i.d., 0.25 mm; film thickness, 0.25 μm; Agilent Technologies, Inc.). Helium was used as the carrier gas at 1 mL/min. The GC temperature was initially maintained at 35 °C for 5 min and then increased to 140 °C at a rate of 4 °C/min for 5 min, heated to 250 °C at a rate of 10 °C/min, and, finally, held at 250 °C for 5 min. The MSD was made according to the manufacturer’s recommendations in the full scan mode. The ion source and transfer line temperatures were 230 °C and 250 °C, respectively. The mass spectra from each sample were recorded using a scan range of 40–400 m/z with electron impact mode set at a voltage of 70 eV.

Volatile compounds were identified by comparing their mass spectra and retention times with those in the National Institute of Standards and Technology database (NIST version 11 mass spectral database; Agilent Technologies Inc.). The retention indexes (RIs) of detected compounds were calculated by injection of a standard mixture containing C3–C25 *n*-alkanes in pure hexane under the same chromatographic conditions and then compared with the RI in the database (http://webbook.nist.gov/chemistry). Each sample measurement was carried out in triplicate.

### 3.6. Statistical Analysis

PCA is commonly used for complex data analysis to summarize variation. It provides a way to characterize multidimensional data and identify similarities and differences. In the present study, the PCA of the data was performed using the SPSS for Windows statistical software package (SPSS Inc., Chicago, IL, USA). Figures were drawn using Origin 7.5 software (OriginLab, Northampton, MA, USA).

## 4. Conclusions

The increasing demand for flavor in various industrial applications has prompted a great deal of interest in the effects of different ratios of *L. delbrueckii* subsp. *bulgaricus* to *S. thermophilus* in fermented milk products. In the present study, 89 volatile flavor compounds were identified using the HS-SPME-GC-MS technique with DVB/Carboxen/PDMS. These included aldehydes, ketones, acids, alcohols, esters, alcohols, and aromatic compounds. There were significant changes in the profiles of volatile flavor compounds depending on the ratio of the initial proportion of *L. delbrueckii* subsp. *bulgaricus* IMAU20401 to that of *S. thermophilus* ND03. In particular, aldehyde and ketone compound concentrations were higher in the 1:1000 treatment combination than in the other combinations. Our results indicated that selecting the appropriate proportions of *L. delbrueckii* subsp. *bulgaricus* and *S. thermophilus* for the starter culture is important for determination of the final profile of volatiles and overall flavor of milk products.

## Figures and Tables

**Table 1:** Volatile compounds produced by milk fermented with different proportions of *L.* *delbrueckii* subsp. *bulgaricus* IMAU20401 and *S. thermophilus* ND03.

No.	Volatile Compound	Chemical Formula	RT (min) ^1^	RI ^2^	RI ^3^	Method ^4^	Ratio of *L. delbrueckii* subsp. *bulgaricus* to *S. thermophiles* (μg/L)					
							1:1	1:10	1:50	1:100	1:1000	1:10,000
**Aldehyde Compounds**												
1	Acetaldehyde	C_2_H_4_O	1.26	-	-	MS, STD	6.86	7.11	7.77	11.15	14.33	-
2	3-Hydroxybutanal	C_4_H_8_O_2_	2.37	605	-	MS	-	1.33	2.39	1.16	6.96	5.46
3	3-Methylbutanal	C_5_H_10_O	2.82	639	634	MS, RI	-	5.55	5.38	8.23	7.80	5.31
4	Pentanal	C_5_H_10_O	3.19	668	668	MS, RI	-	-	0.32	1.36	1.07	-
5	(*E*)-2-Pentenal	C_5_H_8_O	5.14	753	754	MS, RI	-	3.89	-	1.27	5.44	-
6	Hexanal	C_6_H_12_O	5.61	768	769	MS, RI	-	1.43	1.29	2.51	5.20	1.24
7	(*E*)-2-Hexenal	C_6_H_10_O	8.35	848	848	MS, RI	2.58	2.01	1.95	2.29	-	-
8	Heptanal	C_7_H_14_O	8.66	857	860	MS, RI	1.55	1.95	1.66	1.86	2.23	-
9	Benzaldehyde	C_7_H_6_O	11.77	932	933	MS, RI	-	-	0.31	-	-	3.05
10	(*E*)-2-Heptenal	C_7_H_12_O	12.28	953	953	MS, RI	3.82	-	-	-	-	0.23
11	(*E*,*E*)-2,4-Heptadienal	C_7_H_10_O	14.23	1006	1007	MS, RI	-	-	1.32	-	-	-
12	(*E*)-2-Octenal	C_8_H_14_O	15.82	1054	1055	MS, RI	1.26	-	0.32	-	-	-
13	Nonanal	C_9_H_18_O	16.85	1085	1086	MS, RI	2.92	0.25	0.31	0.30	0.86	1.22
14	(*E*)-2-Nonenal	C_9_H_16_O	19.03	1155	1157	MS, RI	3.93	-	-	-	-	-
15	Decanal	C_10_H_20_O	19.91	1184	1185	MS, RI	1.93	-	-	-	0.67	-
16	(*Z*)-2-Decenal	C_10_H_18_O	22.01	1257	1252	MS, RI	1.26	-	**-**	-	-	-
**Ketone Compounds**												
17	2,3-Butanedione	C_4_H_6_O_2_	2.25	-	-	MS, STD	-	1.06	2.11	2.33	2.13	5.88
18	2-Pentanone	C_5_H_10_O	2.97	651	653	MS, RI	-	-	3.89	1.67	5.27	5.04
19	3-Methyl-2-butanone	C_5_H_10_O	3.01	654	654	MS, RI	1.65	3.26	3.45	4.21	5.28	2.61
20	1-Hydroxy-2-propanone	C_3_H_6_O_2_	3.33	679	674	MS, RI	-	5.23	-	-	-	-
21	Acetoin	C_4_H_8_O_2_	3.42	686	-	MS, STD	-	41.18	87.38	94.26	111.89	68.57
22	3-Methyl-(*S*)-2-butanol	C_5_H_12_O	5.35	760	-	MS	-	-	-	-	-	4.30
23	2-Heptanone	C_7_H_14_O	9.23	871	863	MS, RI	21.79	9.00	17.18	11.27	25.56	27.48
24	5-Methyl-3-heptanone	C_8_H_16_O	12.43	957	962	MS, RI	1.31	-	-	-	-	-
25	2-Propyl-1-heptanol	C_10_H_22_O	15.29	1033	-	MS, RI	-	-	0.25	0.18	-	-
26	2,5-Dimethyl-4-hydroxy-3(2*H*)-furanone	C_6_H_8_O_3_	16.08	1062	1060	MS, RI	-	0.53	-	-	-	-
27	2-Nonanone	C_9_H_18_O	16.45	1071	1070	MS, RI	14.04	2.11	5.43	5.84	10.67	6.77
28	2,3-Dihydro-3,5-dihydroxy-6-methyl-4*H*-pyran-4-one	C_6_H_8_O_4_	18.56	1140	1149	MS, RI	-	0.70	-	-	-	-
29	2-Undecanone	C_11_H_22_O	22.48	1273	1273	MS, RI	1.84	0.85	1.07	0.62	1.39	1.03
**Acid Compounds**												
30	Acetic acid	C_2_H_4_O_2_	2.56	619	622	MS, RI, STD	24.65	18.48	17.08	14.48	13.28	7.89
31	2-Oxopropanoic acid	C_3_H_4_O_3_	3.08	659	-	MS	-	1.70	1.34	1.18	1.67	1.79
32	2-Methylpropanoic acid	C_4_H_8_O_2_	5.12	752	753	MS, RI	2.88	5.68	6.64	5.32	4.85	-
33	Butanoic acid	C_4_H_8_O_2_	6.30	793	793	MS, RI	16.83	12.07	11.40	11.14	4.02	0.62
34	3-Methylbutanoic acid	C_5_H_10_O_2_	8.22	845	845	MS, RI	-	3.35	8.33	6.79	5.04	4.09
35	2-Methylhexanoic acid	C_7_H_14_O_2_	8.93	863	-	MS	-	5.31	8.15	5.33	2.74	3.60
36	2-Methylbutanoic acid	C_5_H_10_O_2_	9.10	868	-	MS	-	7.16	12.42	10.24	9.29	-
37	Pentanoic acid	C_5_H_10_O_2_	9.27	872	875	MS, RI	-	10.27	11.86	11.03	8.38	-
38	Lactic acid	C_3_H_6_O_3_	10.82	914	-	MS, STD	6.67	5.41	-	-	-	-
39	Hexanoic acid	C_6_H_12_O_2_	13.16	974	974	MS, RI	40.57	34.38	30.15	35.00	25.29	22.88
40	7-Oxo-Octanoic acid	C_8_H_14_O_3_	13.56	987	-	MS	3.94	-	-	-	-	-
41	Heptanoic acid	C_7_H_14_O_2_	16.77	1083	1085	MS, RI	2.02	1.98	1.29	1.04	-	-
42	Octanoic acid	C_8_H_16_O_2_	19.70	1177	1178	MS, RI	13.43	7.85	10.13	6.08	12.43	8.11
43	Nonanoic acid	C_9_H_18_O_2_	21.08	1224	1226	MS, RI	-	2.99	1.85	1.69	1.78	-
44	2-Undecenoic acid	C_11_H_20_O_2_	22.31	1267	-	MS	0.73	0.67	-	0.35	1.11	-
45	*n*-Decanoic acid	C_10_H_20_O_2_	24.54	1349	1349	MS, RI	2.01	1.74	3.66	2.40	1.07	3.79
**Ester Compounds**												
46	Formic acid, hexyl ester	C_7_H_14_O_2_	8.94	864	870	MS, RI	1.44	1.89	1.97	2.65	1.87	2.29
47	Heptanoic acid, 2-methyl-2-butyl ester	C_12_H_24_O_2_	14.10	1002	-	MS	-	-	-	-	0.89	-
48	Sec-butyl nitrite	C_4_H_9_NO_2_	17.02	1090	-	MS	-	1.27	-	-	-	-
49	Allyl 2-ethyl butyrate	C_9_H_16_O_2_	20.39	1200	-	MS	-	-	-	0.73	1.50	-
50	Butanoic acid, 2-ethyl-,1,2,3-propanetriyl ester	C_21_H_38_O_6_	20.40	1200	-	MS	-	-	1.48	1.49	1.94	-
51	Pentanoic acid, heptyl ester	C_12_H_24_O_2_	25.22	1375	1376	MS, RI	-	0.14	-	1.93	-	-
**Alcohol Compounds**												
52	Cyclobutanol	C_4_H_8_O	1.81	-	-	MS	-	4.22	5.49	6.49	-	-
53	Trans-4-methylcyclohexanol	C_7_H_14_O	3.18	667	-	MS	9.57	-	-	-	-	-
54	3-Methyl-2-butanol	C_5_H_12_O	3.38	682	700	MS, RI	3.92	4.69	5.03	4.99	-	-
55	3-Methyl-3-buten-1-ol	C_5_H_10_O	3.53	694	726	MS, RI	-	-	-	-	4.92	-
56	3-Methylbutanol	C_5_H_12_O	4.54	732	732	MS, RI	-	23.12	20.55	14.89	15.86	-
57	2,2-Dimethyl-1-butanol	C_6_H_14_O	4.83	742	-	MS	-	-	-	-	3.06	-
58	Trans-1,2-cyclopentanediol	C_5_H_10_O_2_	5.49	765	-	MS	-	-	-	-	1.73	0.92
59	1-Propoxy-2-propanol	C_6_H_14_O_2_	5.67	771	-	MS	-	-	-	-	-	1.13
60	2-Methyl-3-pentanol	C_6_H_14_O	6.67	804	805	MS, RI	-	-	2.09	-	-	-
61	1-Hexanol	C_6_H_14_O	8.50	852	858	MS, RI	16.29	7.45	6.92	5.90	3.05	5.54
62	2-Methyl-3-pentanol	C_8_H_18_O	9.40	876	-	MS	-	1.66	12.14	-	-	-
63	3-Methyl-2-hexanol	C_7_H_16_O	10.30	900	906	MS, RI	-	0.24	1.96	2.96	-	-
64	5-Methyl-2-heptanol	C_8_H_18_O	11.26	925	-	MS	-	0.71	-	-	-	-
65	2-Heptanol	C_7_H_16_O	11.47	931	915	MS, RI	-	0.93	-	-	-	0.42
66	1-Heptanol	C_7_H_16_O	13.17	976	974	MS, RI	12.49	1.86	2.92	3.10	8.37	4.38
67	1-Octen-3-ol	C_8_H_16_O	13.26	979	979	MS, RI	2.15	-	-	-	-	-
68	3,5-Octadien-2-ol	C_8_H_14_O	15.21	1036	1037	MS, RI	0.82	-	-	-	-	-
69	2-Nonen-1-ol	C_9_H_18_O	15.79	1053	-	MS	-	3.46	1.93	1.42	2.33	2.58
70	(*E*)-2-Octen-1-ol	C_8_H_16_O	16.21	1066	1067	MS, RI	1.24	-	-	-	-	-
71	5-Ethyl-2-heptanol	C_9_H_20_O	16.76	1082	-	MS	-	-	-	-	1.16	-
72	2-Nonanol	C_9_H_20_O	17.24	1097	1098	MS, RI	1.14	1.59	2.38	3.98	-	-
73	(*E*)-2-Nonen-1-ol	C_9_H_18_O	19.32	1165	1171	MS, RI	1.26	-	-	-	-	-
74	1-Nonanol	C_9_H_20_O	19.41	1168	1168	MS, RI	7.45	6.39	4.92	6.12	5.29	6.21
75	(*E*)-2-Decen-1-ol	C_10_H_20_O	22.23	1265	-	MS	1.64	-	-	-	-	-
76	2-Undecanol	C_11_H_24_O	23.15	1297	1303	MS, RI	-	0.62	-	0.28	-	-
**Aromatic Hydrocarbons**												
77	Toluene	C_7_H_8_	5.08	751	757	MS, RI	-	0.23	5.13	4.16	3.45	-
78	1,3,5-Cycloheptatriene	C_7_H_8_	5.38	761	765	MS, RI	-	-	-	-	-	0.91
79	2,4-Dimethylhexane	C_8_H_18_	6.15	787	-	MS	7.39	1.13	-	-	-	-
80	1-Octene	C_8_H_16_	6.17	788	791	MS, RI	1.35	0.98	-	-	-	-
81	(*E*)-5-Methyl-2-hexene	C_7_H_14_	8.48	852	-	MS	-	-	-	1.52	-	-
82	(*Z*)-2-Heptene	C_7_H_14_	9.17	870	-	MS	-	4.84	-	-	-	-
83	Dodecane	C_12_H_26_	15.29	1038	-	MS	-	-	-	2.04	-	0.69
84	1-Nonyne	C_9_H_16_	15.78	1049	-	MS	-	1.91	1.89	2.17	-	-
85	2,4,6-Trimethyldecane	C_13_H_28_	17.91	1118	1121	MS, RI	-	-	1.54	1.62	1.65	-
86	4,6-Decadiene	C_10_H_18_	17.92	1119	-	MS	2.56	-	-	-	-	-
87	4-Ethylphenol	C_8_H_10_O	19.12	1158	1161	MS, RI	0.64	-	-	-	-	-
88	2-Methylundecane	C_12_H_26_	19.33	1162	1164	MS, RI	-	-	-	1.66	-	-
89	2,3,5,8-Tetramethyldecane	C_14_H_30_	21.85	1251	-	MS	-	0.32	0.48	0.36	0.96	-

^1^ Retention time; ^2^ Retention index of unknown compounds on an HP-5MS column calculated against the gas chromatography-mass spectrometry (GC-MS) retention time of *n*-alkanes (C3–C25); ^3^ RI from database (http://webbook.nist.gov/chemistry); ^4^ RI, agreed with retention index in the literature; MS, compared with NIST 11 Mass Spectral Database; STD, agreed with the mass spectrum of standard chemical. ‘-’ = not detected.

## References

[1] Bernardeau M., Vernoux J.P., Henri-Dubernet S., Gueguen M. (2008). Safety assessment of dairy microorganisms: The *Lactobacillus* genus. Int. J. Food Microbiol..

[2] Yu J., Sun Z., Liu W., Xi X., Song Y., Xu H., Lv Q., Bao Q., Menghe B., Sun T. (2015). Multilocus sequence typing of *Streptococcus thermophilus* from naturally fermented dairy foods in China and Mongolia. BMC Microbiol..

[3] Suzuki I., Kato S., Kitada T., Yano N., Morichi T. (1986). Growth of *Lactobacillus bulgaricus* in Milk. 1. Cell Elongation and the Role of Formic Acid in Boiled Milk. J. Dairy Sci..

[4] Sieuwerts S., Bok F.A.M.D., Hugenholtz J., Vlieg J.E.T.V.H. (2008). Unraveling microbial interactions in food fermentations: From classical to genomics approaches. Appl. Environ. Microbiol..

[5] Kaneko D., Igarashi T., Aoyama K. (2014). Reduction of the off-flavor volatile generated by the yogurt starter culture including *Streptococcus thermophilus* and *Lactobacillus delbrueckii* subsp. *bulgaricus* in soymilk. J. Agric. Food Chem..

[6] Beshkova D.M., Simova E.D., Frengova G.I., Simov Z.I., Dimitrov Z.P. (2003). Production of volatile aroma compounds by kefir starter cultures. Int. Dairy J..

[7] Lin J., Hua B., Xu Z., Li S., Ma C. (2016). The impact of proteolytic pork hydrolysate on microbial, flavor and free 388 amino acids compounds of yogurt. Korean J. Food Sci. Anim. Resour..

[8] Settachaimongkon S., Nout M.J., Antunes Fernandes E.C., Hettinga K.A., Vervoort J.M., van Hooijdonk T.C., Zwietering M.H., Smid E.J., van Valenberg H.J. (2014). Influence of different proteolytic strains of *Streptococcus thermophilus* inco-culture with *Lactobacillus delbrueckii* subsp. *bulgaricus* on themetabolite profile of set-yoghurt. Int. J. Food Microbiol..

[9] Dan T., Wang D., Jin R.L., Zhang H.P., Zhou T.T., Sun T.S. (2017). Characterization of volatile compounds in fermented milk using solid-phase microextraction methods coupled with gas chromatography-mass spectrometry. J. Dairy Sci..

[10] Arthur C.L., Pawliszyn J. (1990). Solid phase microextraction with thermal desorption using fused silica optical fibers. Anal. Chem..

[11] Chiofalo B., Zumbo A.R., Liotta L., Mondello L., Dugo P., Chiofalo V. (2004). Characterization of Maltese goat milk cheese flavour using SPME-GC/MS. S. Afr. J. Anim. Sci..

[12] Yang X., Peppard T. (1994). Solid-phase microextraction for flavor analysis. J. Agric. Food Chem..

[13] Lubbers S., Decourcelle N., Vallet N., Guichard E. (2004). Flavor release and rheology behavior of strawberry fat free stirred yogurt during storage. J. Agric. Food Chem..

[14] Li N., Zheng F.-P., Chen H.-T., Liu S.-Y., Chen G., Song Z.-Y., Sun B.-G. (2011). Identification of volatile components in chinese sinkiang fermented camel milk using SAFE, SDE, and HS-410 SPME-GC/MS. Food Chem..

[15] San A.T., Joyce D.C., Hofman P.J., Macnish A.J., Webb R.I., Matovic N.J., Williams C.M., Voss J.J.D., Wong S.H., Smyth H.E. (2017). Stable isotope dilution assay (SIDA) and HS-SPME-GCMS quantification of key aroma volatiles for fruit and sap of Australian mango cultivars. Food Chem..

[16] Panighel A., Flamini R. (2014). Applications of solid-phase microextraction and gas chromatography/mass spectrometry (SPME-GC/MS) in the study of grape and wine volatile compounds. Molecules.

[17] Corral S., Leitner E., Siegmund B., Flores M. (2016). Determination of sulfur and nitrogen compounds during the processing of dry fermented sausages and their relation to amino acid generation. Food Chem..

[18] Rodrigues F., Caldeira M., Câmara J.S. (2008). Development of a dynamic headspace solid-phase microextraction procedure coupled to GC-qMSD for evaluation the chemical profile in alcoholic beverages. Anal. Chim. Acta.

[19] Sun Z.H., Chen X., Wang J.C., Zhao W.J., Shao Y.Y., Wu L., Zhou Z.M., Sun T.S., Wang L., Meng H., Zhang H.P., Chen W. (2011). Complete genome sequence of *Streptococcus thermophilus* strain ND03. J. Bacteriol..

[20] Zha M., Yu J., Zhang Y., Wang H., Bai N., Qin Y., Liangliang D., Liu W., Zhang H., Bilige M. (2015). Study on *Streptococcus thermophilus* isolated from Qula and associated characteristic of acetaldehyde and 432 diacetyl in their fermentedmilk. J. Gen. Appl. Microbiol..

[21] Chaves A.C.S.D., Fernandez M., Lerayer A.L.S., Mierau I., Kleerebezem M., Hugenholtz J. (2002). Metabolic engineering of acetaldehyde production by *Streptococcus thermophilus*. Appl. Environ. Microbiol..

[22] Wang M., Mcintee E.J., Cheng G., Shi Y., Villalta P.W., Hecht S.S. (2000). Identification of DNA adducts of acetaldehyde. Chem. Res. Toxicol..

[23] Cheng H. (2010). Volatile flavor compounds in yogurt: A review. Crit. Rev. Food Sci. Nutr..

[24] Ott A., Germond J.E., Germond A. (2000). Vicinal diketone formation in yogurt: 13C precursors and effect of branched-chain amino acids. J. Agric. Food Chem..

[25] Ramya I., Tomar S.K., Uma M.T., Rameshwar S. (2010). *Streptococcus thermophilus* strains: Multifunctional lactic acid bacteria. Int. Dairy J..

[26] Hamdan I.Y., Kunsman J.E., Deanne D.D. (1971). Acetaldehyde production by combined yogurt cultures. J. Dairy Sci..

[27] Valero E., Villamiel M., Miralles B., Sanz J., MartíNez-Castro I. (2001). Changes in flavour and volatile components during storage of whole and skimmed UHT milk. Food Chem..

[28] Gadaga T.H., Viljoen B.C., Narvhus J.A. (2007). Volatile organic compounds in naturally fermented milk and milk fermented using yeasts, lactic acid bacteria and their combinations as starter cultures. Food Technol. Biotechnol..

[29] Sheldon R.M., Lindsay R.C., Libbey L.M., Morgan M.E. (1971). Chemical nature of malty flavor and aroma produced by *Streptococcus lactis* var. *maltigenes*. Appl. Microbiol..

[30] Liang H.Z., Yang S.P., Liu W.J., Yuan L.I. (2012). In situ real-time monitoring of volatile metabolites of fermented milk by dynamic headspace sampling-atmospheric pressure ionization mass spectrometry. Food Sci..

[31] Fortini M., Migliorini M., Cherubini C., Cecchi L., Calamai L. (2017). Multiple internal standard normalization for improving HS-SPME-GC-MS quantitation in virgin olive oil volatile organic compounds (VOO-VOCs) profile. Talanta.

[32] Alemayehu D., Hannon J.A., Mcauliffe O., Ross R.P. (2014). Characterization of plant-derived lactococci on the basis of their volatile compounds profile when grown in milk. Int. J. Food Microbiol..

[33] Pan D.D., Wu Z., Peng T., Zeng X.Q., Li H. (2014). Volatile organic compounds profile during milk fermentation by *Lactobacillus pentosus* and correlations between volatiles flavor and carbohydrate metabolism. J. Dairy Sci..

[34] Rincon-Delgadillo M.I., Lopez-Hernandez A., Wijaya I., Rankin S.A. (2012). Diacetyl levels and volatile profiles of commercial starter distillates and selected dairy foods. J. Dairy Sci..

[35] Bok F.A.M.D., Janssen P.W.M., Bayjanov J.R., Sieuwerts S., Lommen A., Vlieg J.E.T.V.H., Molenaar D. (2011). Volatile compound fingerprinting of mixed-culture fermentations. Appl. Environ. Microbiol..

[36] Pereda J., Jaramillo D.P., Quevedo J.M., Ferragut V., Guamis B., Trujillo A.J. (2008). Characterization of volatile compounds in ultra-high-pressure homogenized milk. Int. Dairy J..

[37] Jeon B.S., Moon C., Kim B.C., Kim H., Um Y., Sang B.I. (2013). In situ extractive fermentation for the production of hexanoic acid from galactitol by *Clostridium sp.* BS-1. Enzym. Microb. Technol..

[38] Güler Z. (2007). Changes in salted yoghurt during storage. Int. J. Food Sci. Technol..

[39] Hettinga K.A., van Valenberg H.J., Lam T.J., van Hooijdonk A.C. (2008). Detection of mastitis pathogens by analysis of volatile bacterial metabolites. J. Dairy Sci..

[40] Smit B.A., Engels W.J., Smit G. (2009). Branched chain aldehydes: Production and breakdown pathways and relevance for flavour in foods. Appl. Microbiol. Biotechnol..

[41] Molimard P., Spinnler H.E. (1996). Review: Compounds involved in the flavor of surface mold-ripened cheeses: Origins and properties. J. Dairy Sci..

[42] Van A.M., Duncan S.E., Marcy J.E., Long T.E., O’Keefe S.F., Nielsen-Sims S.R. (2005). Aroma analysis of light-exposed milk stored with and without natural and synthetic antioxidants. J. Dairy Sci..

[43] Moinas M., Groux M., Horman I. (1975). Flaveur des fromages. iii. mise en evidencede quelques constituants mineurs de l’arome du camembert. Dairy Sci. Technol..

[44] Friedrich J.E., Acree T.E. (1998). Gas chromatography olfactometry (GC/O) of dairy products. Int. Dairy J..

[45] Fuchs G. (2008). Anaerobic metabolism of aromatic compounds. Ann. N. Y. Acad. Sci..

[46] Aunsbjerg S.D., Honoré A.H., Marcussen J., Ebrahimi P., Vogensen F.K., Benfeldt C., Skov T., Knøchel S. (2015). Contribution of volatiles to the antifungal effect of *Lactobacillus paracasei* in defined medium and yogurt. Int. J. Food Microbiol..

